# Mirtazapine Risk of Hyponatremia and Syndrome of Inappropriate Antidiuretic Hormone Secretion in Adult and Elderly Patients: A Systematic Review

**DOI:** 10.7759/cureus.20823

**Published:** 2021-12-30

**Authors:** Alberto Moscona-Nissan, Juan Carlos López-Hernández, Ana P González-Morales

**Affiliations:** 1 Medicine, School of Medicine, Universidad Panamericana, Mexico City, MEX; 2 Neuromuscular Diseases, Instituto Nacional de Neurología y Neurocirugía Manuel Velasco Suárez, Mexico City, MEX

**Keywords:** geriatric psychiatry, hyponatremia, syndrome of inappropriate antidiuretic hormone secretion, antidepressants, mirtazapine

## Abstract

Hyponatremia is the most common fluid and electrolyte imbalance in hospitalized patients. Among hyponatremia causes, syndrome of inappropriate antidiuretic hormone secretion is a condition characterized by excessive release of antidiuretic hormone from the pituitary gland or nonpituitary sources. One of the most common drugs associated with hyponatremia is selective serotonin reuptake inhibitors, especially in elderly patients. Therefore, distinct therapeutic alternatives are essential for patients having risk factors for hyponatremia or syndrome of inappropriate antidiuretic hormone secretion development.

The present article aims to review the available literature evaluating mirtazapine-induced hyponatremia or syndrome of inappropriate antidiuretic hormone secretion in adult or elderly patients in order to determine the incidence of these adverse effects and analyze the existence of any correlation between the administered dose of mirtazapine and serum sodium levels. A systematic search was conducted, using key terms from the research topic, their synonyms, and Boolean/logic operators. From this evidence pool, inclusion and exclusion criteria were applied. We abstracted population characteristics and clinical endpoints. Relevant data from selected studies was abstracted and subject to statistical analysis.

A total sample size of 30,851 patients treated with mirtazapine was included. Mirtazapine-induced hyponatremia incidence was 3.26% (95% CI 3.06-3.45%), with the syndrome of inappropriate antidiuretic hormone secretion (SIADH) the most probable underlying cause. Among case series and case reports evaluated (n=7), hyponatremia and SIADH were more frequent in female patients (71.4%) and the most frequent clinical manifestations included confusion (57%), somnolence (42%), and altered speech (28%). Mean serum sodium levels were (117 mEq/L, ranging from 113-130 mEq/L). The average time lapse between mirtazapine administration and clinical findings was 34 days. The Spearman's rank correlation coefficient between mirtazapine dosage and serum sodium levels was -0.3181 with a p-value >0.05.

In conclusion, mirtazapine presents a moderate risk of hyponatremia and should be considered as an alternative therapy in patients requiring antidepressants with risk factors for this preventable adverse effect.

## Introduction and background

Hyponatremia (serum sodium levels <135 mmol/L) is the most common fluid and electrolyte imbalance in hospitalized patients [1]. It is more frequent among geriatric patients, with an estimated prevalence in hospitalized patients up to 32.5% [1]. The presence of hyponatremia in older patients is associated with impaired cognition, delirium, and a higher incidence of falls and fractures [2]. For this reason, the early identification and treatment of this condition are of great importance. According to serum osmolality, hyponatremia can be classified as hypotonic, isotonic, or hypertonic hyponatremia. In addition, hypotonic hyponatremia can be divided according to the patient's volume status in hypovolemic (caused by diarrhea, dehydration, adrenal insufficiency, diuretics, and vomiting), euvolemic (caused by SIADH, psychogenic polydipsia, or hypothyroidism), and hypervolemic (caused by congestive heart failure, cirrhosis, nephrotic syndrome, or chronic kidney disease) [3].

Syndrome of inappropriate antidiuretic hormone secretion is a condition characterized by excessive release of antidiuretic hormone (ADH) from the pituitary gland or nonpituitary sources [4]. Patients with SIADH present a reduction in water excretion, leading usually to euvolemic hyponatremia. The Bartter and Schwartz criteria for SIADH include serum sodium lower than 135 mEq/L, serum osmolality less than 275 mOsm/kg, urine sodium greater than 40mEq/L, urine osmolality higher than 100 mOsm/kg, absence of clinical evidence of volume depletion, and other underlying causes of hyponatremia [4]. 

Several conditions have been associated with SIADH, such as central nervous system (CNS) alterations (as stroke, hemorrhage, infection, or trauma), malignancies (small cell lung cancer, olfactory neuroblastomas), surgery, pulmonary disease (pneumonia, tuberculosis), and drugs [4]. Several drugs enhance the release of ADH, and, therefore, have been associated with SIADH [4]. 

The most common drugs which cause SIADH include carbamazepine, oxcarbazepine, cyclophosphamide, interferons, haloperidol, methotrexate, and selective serotonin reuptake inhibitors (SSRIs), especially in patients older than 65 years [4]. Although SSRIs are the antidepressant group most linked to SIADH and hyponatremia, other antidepressants such as venlafaxine, when combined with the patient's risk factors, have a high incidence of hyponatremia [5,6]. Mirtazapine risk of SIADH is considered moderate, being an alternative treatment for patients with risk factors [5,6]. 

SSRIs are the antidepressant medication most used in medical practice. This group of drugs works by inhibiting serotonin transporter (SERT), a presynaptic pump that participates by recapturing the serotonin released in the synaptic cleft [7]. For almost a decade, researchers have been investigating the relationship that SSRIs and other antidepressants have with the development of SIADH and, consequently, hyponatremia [5,6]. The mechanism by which SSRIs contribute to the development of SIADH has not been clearly defined. Current theories are based on the results obtained from animal models. In them, it has been seen that increased stimulation, both serotonergic and noradrenergic, contributes to increased ADH secretion [5,6]. 

Certain characteristics have been associated with a higher risk of developing hyponatremia while being treated with SSRIs. It has been found that the risk of developing SIADH in patients treated with SSRIs is higher in women, patients over 65 years of age, and with concomitant use of other drugs capable of contributing to the development of hyponatremia (mainly thiazide diuretics) [5,6]. Hyponatremia in geriatric patients, resulting from the use of SSRIs, is a relatively common adverse reaction. It is important to mention that it is also a frequent cause of hospitalization. This has motivated the search for alternative medications for the treatment of these patients [5,6].

Mirtazapine’s mechanism of action is different from that of other antidepressants. This drug works by increasing the neurotransmission of norepinephrine (NA) and serotonin by inhibiting the α2 adrenergic receptor (presynaptic), resulting in increased intracellular NA concentrations [8]. The increase in NA in serotonergic neurons stimulates α1 adrenoreceptors, thus increasing the release of serotonin towards the synaptic cleft. Mirtazapine also exerts a potent antagonistic effect at the 5HT-2, 5HT-3, and H1 receptors [8]. Through this inhibition, it prevents serotonergic neurons from being overexcited but contributes to the development of sedation (one of its main adverse reactions) [8]. 

Sedation and weight gain are the two most common adverse reactions of mirtazapine. On the other hand, hyponatremia is considered a rare adverse reaction associated with the use of this medication [8]. Mirtazapine has been identified as an antidepressant with a low risk of contributing to the development of hyponatremia (<1%). In comparison, the incidence of hyponatremia related to the use of SSRIs is much higher (1-32%) [9].

The objective of this article is to systematically review the available literature (experimental or observational studies) evaluating mirtazapine-induced hyponatremia or SIADH in adult or elderly patients in order to establish a correlation and determine the incidence of these adverse effects. Additionally, analyze the existence of any correlation between the administered dose of mirtazapine and serum sodium levels. 

## Review

Methods

The literature published before November 2021 was searched by conducting a systematic search in PubMed, Scopus, and grey literature restricted to English, Portuguese and Spanish languages. Using key terms from the research topic, we developed a search matrix, including control vocabulary, such as MESH terms “Mirtazapine”, “Hyponatremia”, their synonyms, and Boolean/logic operators. From this evidence pool, inclusion and exclusion criteria were applied and duplicate articles eliminated. 

Inclusion criteria included: 1) experimental studies (randomized controlled trials, natural experiments) and/or, 2) observational studies (case-control, cohort, cross-sectional, case reports), 3) evaluating mirtazapine-induced hyponatremia or SIADH, 4) English, Portuguese, or Spanish full-text availability, 5) patients ≥ 18 years old, 6) incidence rate ratio or hyponatremia incidence parameters availability. Exclusion criteria included 1) patients <18 years old, 2) underlying patient comorbidities that confuse mirtazapine and hyponatremia correlation, 3) no full-text availability or written in languages different to English, Spanish, or Portuguese, 4) incidence rate ratio or hyponatremia incidence parameters unavailable.

**Figure 1:**
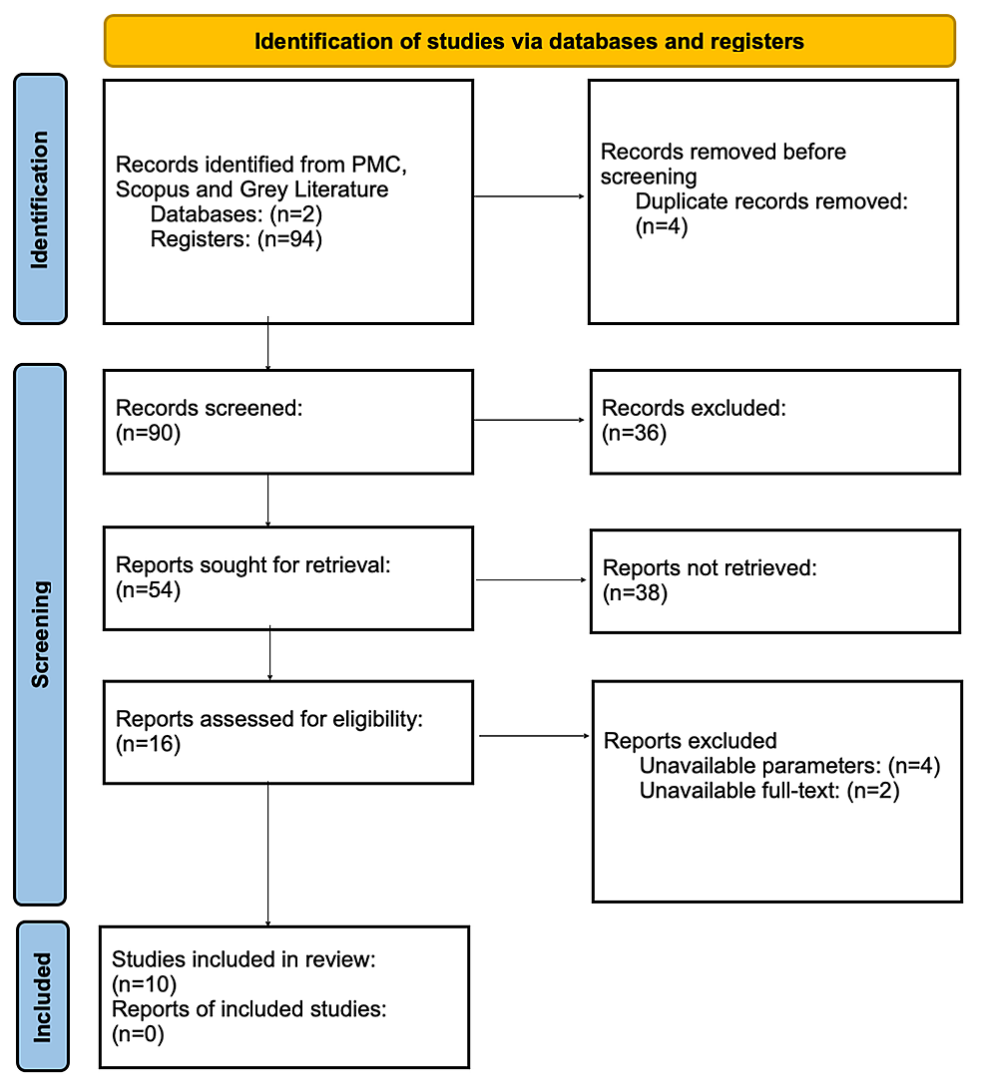
PRISMA flow diagram PRISMA: Preferred Reporting Items for Systematic Reviews and Meta-Analyses

As shown in Figure 1, the initial pool of evidence comprised 94 articles, of which four were duplicate results. The 90 remaining articles were screened by assessment of their titles and abstracts. Papers addressing other topics were dismissed, discarding 36 articles. The remaining 54 papers were subjected to an evaluation of whether they met inclusion criteria or not. After completing so and assessing eligibility, 10 studies were finally selected (Figure 1).

Due to broad inclusion criteria, considerable heterogeneity was found among selected studies. Therefore, we abstracted population (mean age, the proportion of females/males) and study characteristics (single vs. multicenter, study design, etc.) and clinical endpoints (hyponatremia levels and incidence) considering possible confounding variables. Data from selected studies was abstracted and presented in the evidence table shown in the results section. Relevant data was extracted from the articles and subject to statistical analysis, estimating the overall incidence of hyponatremia and SIADH, calculating the mean, standard deviation (SD), and 95% confidence interval (CI) of certain values as serum sodium level, mean dosage and the time gap between mirtazapine administration and hyponatremia. The frequency of certain clinical manifestations of hyponatremia and other abnormal laboratory findings were estimated from the available data. Finally, in order to determine the existence of a correlation between a higher dose of mirtazapine and decreased serum sodium levels, Spearman's rank correlation coefficient and its p-value were calculated. A nonparametric test was applied due to the lack of normal distribution of data and a small sample size. 

Results

Our literature search identified 10 eligible articles (Table 1) published in eight different countries from 2003 to 2016. Two retrospective cohorts, a case-control study, a case series, and six case reports were included. The total sample size was 30,851 patients treated with mirtazapine, of which 43.94% were male and 56.06% were female. The patients' mean age was 49.02 years.

**Table 1:** Qualitative and quantitative aspects of included studies (author, year, country, study type and demographics) NS = Not Specified

Author	Year	Country	Type of study	Sample Size	Male (%)	Female (%)	Mean age (years)
Degner et al. [10]	2004	Germany	Retrospective Cohort	4750	NS	NS	NS
Jung et al. [11]	2011	Korea	Retrospective Cohort	76	23.7	62.4	52.9
Grover et al. [12]	2014	India	Case-control	15	53.8	46.2	66.8
Leth-Møller et al. [13]	2016	Denmark	Retrospective Cohort	26003	44	56	49
Bavbek et al. [14]	2006	Turkey	Case report	1	0	100	67
Cheah et al. [15]	2008	Australia	Case series	2	50	50	70
Ladino et al. [16]	2006	USA	Case report	1	100	0	72
Roxanas et al. [17]	2003	Australia	Case report	1	0	100	86
Famularo et al. [18]	2009	Italy	Case report	1	0	100	76
Ghosh et al. [19]	2014	India	Case report	1	0	100	46

Among retrospective cohorts and case-control studies, 1,007 of 30,844 patients treated with mirtazapine developed hyponatremia, the incidence being 3.26%. The calculated standard error (SE) of the proportion was 0.001 and 95% CI for the proportion was 3.06-3.45. Additional reported adverse effects in the studies included elevated transaminases, cardiovascular alterations, cutaneous edema, subclinical pancreatitis, and restless legs (Table 2). 

**Table 2:** Cohort and Case-control studies clinical outcomes NS = Not Specified; IRR = Incidence Rate Ratio

Author	Clinical manifestations	Other reported adverse effects	Outcome (Incidence, IRR)
Degner et al. [10]	None	Hepatic (elevated transaminases), Cardiovascular, dermatological (cutaneous edema), restless legs, subclinical pancreatitis	0%, NS
Jung et al. [11]	None	NS	0%, NS
Grover et al. [12]	NS	NS	80%, NS
Leth-Møller et al. [13]	NS	NS	3.84%, 1.12 [1.05-1.19]

Case series and case report studies (Table 3), evaluated a total of seven patients who developed hyponatremia after mirtazapine intake, of which five were female (71.42%) and two male (28.57%). The most prevalent clinical manifestations found were confusion in 4/7 patients (57.14%), somnolence in 3/7 cases (42.85%), and altered speech in 2/7 patients (28.57%). Other clinical manifestations included nausea, vomit, agitation, irritation, and impairment of short-term memory. 

**Table 3:** Case series and case report studies clinical outcomes: descriptive characteristics of mirtazapine induced hyponatremia cases NS = Not Specified; BID = twice a day; ADH = antidiuretic hormone

Author	Clinical manifestations	Serum sodium levels (mEq/L)	Previous medication	Mirtazapine dosage	Time gap between administration and hyponatremia (days)	Abnormal laboratory findings	Management
Bavbek et al. [14]	2-day history of nausea and intermittent vomiting. Patient was afebrile, with normal vital signs	115	Levodopa and citalopram, 20 mg/d, 7 months prior to treatment	15 mg nightly	153	Urine sodium of 249 mEq/L, serum osmolarity of 265 mOsm/kg and urine osmolarity of 386 mOsm/kg.	Mirtazapine therapy was discontinued, treatment with hypertonic saline at 2 mEq/L/h, tianeptine prescription when discharged.
Cheah et al. [15]	Somnolence	112	Metformin 500 mg BID, gliclazide 40 mg BID, irbesartan 150 mg daily, and hydrochlorothiazide 12.5 mg daily	15 mg daily	7	Urine osmolarity of 353 mOsm/kg; serum osmolarity of 239 mOsm/kg.	Mirtazapine therapy was discontinued, treatment with hypertonic saline, followed by fluid restriction.
Cheah et al. [15]	Confusion	113	Ramipril 5 mg daily, metoprolol 25 mg BID, furosemide 40 mg daily	30 mg daily	10	Urine osmolarity of 320 mOsm/kg; serum osmolarity of 243 mOsm/kg.	Mirtazapine and furosemide were discontinued, fluid restriction.
Ladino et al. [16]	Confusion and somnolence	116	NS	7.5 mg nightly	6	serum osmolarity of 254 mOsm/kg; serum potassium of 5.9 mmol/L.	Mirtazapine therapy was discontinued
Roxanas et al. [17]	NS	130	Venlafaxine, amiodarone, gliclazide, L-thyroxine, irbesartan, hydrochlorothiazide, alendronate, omeprazole, atorvastatin and zolpidem	15 mg nightly	4	Urine osmolarity of 398 mOsm/kg; serum osmolarity of 294 mOsm/kg.	Mirtazapine therapy was discontinued, ADH serum level of 0.7 pmol/L
Famularo et al. [18]	Lethargy, confusion, somnolence and dysphasia	114	Telmisartan and hydrochlorothiazide	30 mg daily	58	Urine osmolarity of 450 mOsm/kg; serum osmolarity of 236 mOsm/kg	Mirtazapine therapy was discontinued, treatment with hypertonic saline.
Ghosh et al. [19]	Confusion, agitation, irritation, altered speech, poor attention, impairment of short-term memory	123	Clonazepam 0.5 mg/day	22.5 mg daily	1	None	Mirtazapine therapy was discontinued, treatment with isotonic saline.

Registered serum sodium levels ranged from 113 to 130 mEq/L. Mean serum sodium levels were 117.57 mEq/L, standard deviation (SD) was 6.06, variance 36.81, 95% CI ranged from 113.08 to 122.06 mEq/L and median serum sodium levels were 115 mEq/L. Regarding mirtazapine dosage among seven cases, the mean dosage was 19.28 mg, ranging from 7.5 to 30 mg of mirtazapine, with an SD of 7.87. The median dosage was 15 mg, having the majority of patients (57.14%) a nightly administration of mirtazapine. 

The average time gap between mirtazapine administration and clinical findings was 34.14 days, ranging from 1 day to 5 months, calculated SD was 51.84. Other abnormal laboratory findings reported were serum osmolarity less than 275 mOsm/kg in 6/7 patients (85.71%), urine osmolarity greater than 100 mOsm/kg in 5/7 patients (71.42%), which are part of Bartter-Schwartz criteria for SIADH [4]. Hyponatremia management consisted in all cases in mirtazapine discontinuation and fluid therapy with hypertonic saline in most cases. 

The Spearman's rank correlation coefficient was calculated in order to evaluate a possible correlation between mirtazapine dosage and serum sodium levels. The calculated Spearman's Rho was -0.3181, indicating a negative weak correlation. Nevertheless, the calculated p-value was 0.486, which is not statistically significant. 

Discussion

As seen in the results section, mirtazapine has a moderate risk of hyponatremia of 3.26%, ranging from 0-80%, among studies. Although the main outcome evaluated was hyponatremia incidence in patients, SIADH represents one of the most probable underlying mechanisms of mirtazapine-induced hyponatremia due to several patients fulfilling several Bartter and Schwartz criteria. 

Certain risk factors should be taken into consideration when prescribing mirtazapine as hospitalization or intensive care unit (ICU) context, elderly patients, female gender, low body mass index (BMI), and concomitant medication with other antidepressants (SSRIs, venlafaxine). Among case series and case reports evaluated in the present review, mirtazapine-induced hyponatremia was found predominantly in female patients (71%). Regarding the dose relation between mirtazapine and sodium levels, a weak negative correlation (inverse relation) was found: lower sodium levels were present at a higher dose, but the results were not statistically significant. 

Given SSRIs and venlafaxine, high rates of hyponatremia, ranging from 0.06-40% and 0.08-70%, respectively. Mirtazapine has been proposed as a therapeutic alternative in elderly and adult patients with risk factors. Several authors (such as Jagsch et al. [20] and Mogi et al. [21]) have reported mirtazapine as a successful therapeutic alternative for patients treated with SSRIs for depression who develop hyponatremia or SIADH.

Jagsch et al. (2007) reported an 81-year-old female treated with citalopram and diagnosed with SIADH. Citalopram was discontinued and replaced with mirtazapine after sodium levels normalization. The patient was prescribed 15 mg daily of mirtazapine for 10 days and 30 mg for more than 60 days [20]. The patient was monitored through laboratory analysis during treatment, finding no abnormalities. Additionally, Mogi et al. (2011), reported a case in which a 65-year-old male was treated with sertraline and presented hyponatremia at day 36 of treatment, sertraline was discontinued and replaced by mirtazapine, managing to correct hyponatremia and treat depression effectively [21]. However, mirtazapine's risk of SIADH and hyponatremia should be estimated with a higher precision in larger cohorts and in patients without concomitant treatment with diuretics and other antidepressants that could cause hyponatremia, which is a limitation of the present study. 

One of the most probable underlying mechanisms of mirtazapine-induced hyponatremia is SIADH. In fact, patients studied in the present review fulfilled Bartter and Schwartz diagnostic criteria in most cases. Serum osmolarity less than 275 mOsm/kg, urine osmolarity greater than 100 mOsm/kg, and urine sodium greater than 40mEq/L were present in most patients, as reported in the results section [4]. Nevertheless, most case reports and case series do not specify that hyponatremia was caused by SIADH. Pathogenic pathways of excessive ADH release secondary to medications have not yet been fully understood. Therefore, further clinical and basic research should be conducted in order to elucidate the mechanisms and risk factors for this preventable adverse effect.

## Conclusions

Mirtazapine presents a moderate risk of hyponatremia and should be considered as an alternative therapy in patients requiring antidepressants with risk factors for hyponatremia or SIADH as elderly, female, low BMI patients and previous SIADH adverse effects on SSRI medication. Hyponatremia represents a potentially dangerous condition that should be suspected and treated in elderly patients developing delirium, somnolence, confusion, agitation, and altered speech. 

The most probable underlying mechanism of mirtazapine-induced hyponatremia is SIADH, fulfilling most patients' Bartter and Schwartz diagnostic criteria. The average time lapse between mirtazapine administration and clinical findings was 34 days, ranging from 1 day to 5 months. Mirtazapine presented a risk of hyponatremia and SIADH of 3.26%, ranging from 0-80%. Nevertheless, mirtazapine's risk of SIADH should be estimated with a higher precision in larger patient cohorts, in order to establish a definitive recommendation. A higher mirtazapine dosage did not show a statistically significant correlation with lower serum sodium levels. Further research should be conducted in order to elucidate the mechanisms of this preventable adverse effect.
